# Reverse mode Na^+^/Ca^2+ ^exchange mediated by STIM1 contributes to Ca^2+ ^influx in airway smooth muscle following agonist stimulation

**DOI:** 10.1186/1465-9921-11-168

**Published:** 2010-12-02

**Authors:** Bo Liu, Samantha E Peel, Jane Fox, Ian P Hall

**Affiliations:** 1 Division of Therapeutics and Molecular Medicine, Respiratory Biomedical Research Unit, Queens Medical Centre, Nottingham, UK

## Abstract

**Background:**

Agonist stimulation of airway smooth muscle (ASM) results in IP_3 _mediated Ca^2+ ^release from the sarcoplasmic reticulum followed by the activation of store operated and receptor operated non-selective cation channels. Activation of these non-selective channels also results in a Na^+ ^influx. This localised increase in Na^+ ^levels can potentially switch the Na^+^/Ca^2+ ^exchanger into reverse mode and so result in a further influx of Ca^2+^. The aim of this study was to characterise the expression and physiological function of the Na^+^/Ca^2+ ^exchanger in cultured human bronchial smooth muscle cells and determine its contribution to agonist induced Ca^2+ ^influx into these cells.

**Methods:**

The expression profile of NCX (which encodes the Na^+^/Ca^2+ ^exchanger) homologues in cultured human bronchial smooth muscle cells was determined by reverse transcriptase PCR. The functional activity of reverse mode NCX was investigated using a combination of whole cell patch clamp, intracellular Ca^2+ ^measurements and porcine airway contractile analyses. KB-R7943 (an antagonist for reverse mode NCX) and target specific siRNA were utilised as tools to inhibit NCX function.

**Results:**

NCX1 protein was detected in cultured human bronchial smooth muscle cells (HBSMC) cells and NCX1.3 was the only mRNA transcript variant detected. A combination of intracellular Na^+ ^loading and addition of extracellular Ca^2+ ^induced an outwardly rectifying current which was augmented following stimulation with histamine. This outwardly rectifying current was inhibited by 10 μM KB-R7943 (an antagonist of reverse mode NCX1) and was reduced in cells incubated with siRNA against NCX1. Interestingly, this outwardly rectifying current was also inhibited following knockdown of STIM1, suggesting for the first time a link between store operated cation entry and NCX1 activation. In addition, 10 μM KB-R7943 inhibited agonist induced changes in cytosolic Ca^2+ ^and induced relaxation of porcine peripheral airways.

**Conclusions:**

Taken together, these data demonstrate a potentially important role for NCX1 in control of Ca^2+ ^homeostasis and link store depletion via STIM1 directly with NCX activation.

## Introduction

The bronchoconstrictor response of the asthmatic airway depends on airway narrowing caused by inappropriate ASM contraction. The ASM layer is also a potential source of pro-inflammatory mediators and so plays a key role in the pathogenesis of this disease. Control of intracellular Ca^2+ ^is critical to regulation of ASM function and mediates many processes including contraction, proliferation and gene expression [1].

Cytoplasmic Ca^2+ ^levels contribute significantly to the contractile/relaxant state of an airway myocyte. Initiation of contraction is dependent upon cytosolic inositol 1, 4, 5-trisphosphate (IP_3_) mediated release of intracellular Ca^2+ ^from the sarcoplasmic reticulum (SR). Sustained contraction is thought to be dependent upon Ca^2+ ^influx from extracellular sources through receptor operated cation (ROC) or store operated cation (SOC) channels on the plasmalemma. The latter are activated following depletion of SR Ca^2+ ^via a mechanism involving STIM1 and ORAI homologues [2,3]. In addition, we have previously shown expression of a range of TRPC homologues in HBSMC [4]. Plasma membrane Na^+^/Ca^2+ ^exchange acting in reverse mode is well documented in cardiac muscle, but only recently has become hypothesised as an additional mechanism of Ca^2+ ^influx in smooth muscle [1,5,6]. Under physiological conditions, Na^+^/Ca^2+ ^exchange is the principle mechanism of Ca^2+ ^extrusion, maintaining low intracellular Ca^2+ ^levels by transporting one Ca^2+ ^in exchange for three Na^+ ^[7]. Under experimental conditions including membrane depolarization and localised increases in intracellular Na^+^, the direction of ion exchange can be reversed resulting in Na^+ ^extrusion and Ca^2+ ^influx. Certain pathophysiological conditions can replicate such conditions including ischemia and exposure to endogenous ouabain [8]. Inhibition of the Na^+^/K^+ ^pump by ouabain, resulting in intracellular Na^+ ^accumulation has been shown to contract human bronchial smooth muscle [9]. In addition, KB-R7943 (a selective antagonist for reverse mode Na^+^/Ca^2+ ^exchange) reduces agonist induced contraction of ASM [1,5,10].

It is clear that reverse mode NCX can be activated following localised intracellular Na^+ ^accumulation, via inhibition of the Na^+^/K^+ ^pump. An hypothesis where Na^+ ^influx through non-selective ROC or SOC channels is sufficient to trigger Ca^2+ ^influx though NCX has recently been proposed [11-13]. NCX1 has also been demonstrated to interact both physically and functionally with certain TRPC homologues [14].

**Figure 1:**
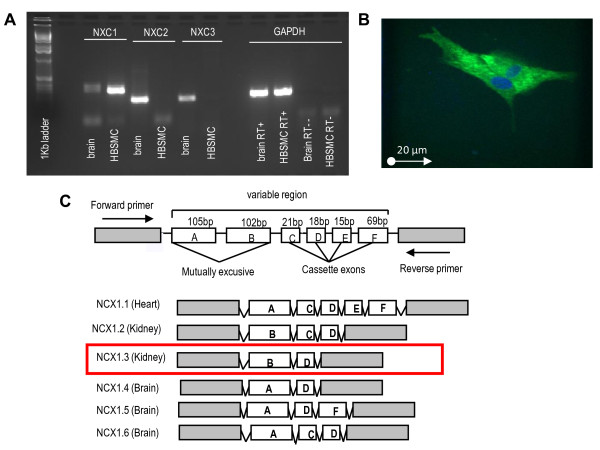
**NCX1 is expressed in cultured HBSMCs**. (A) mRNA expression of NCX1, 2 & 3 in HBSMCs (Human bronchial smooth muscle cells) and human brain. NCX1 mRNA was detected in HBSMCs. NCX2 and NCX3 were expressed in human brain and so used as a positive control. (B) HBSMCs stained with NCX1 antibody after permeabilization followed by labelling with Alexa fluor 48. (C) Gene structure of NCX1 (Adapted from: [15,16]. NCX1.3 transcript also found in the kidney was the only transcript detected in HBSMCs (3 donors).

Mammalian Na^+^/Ca^2+ ^exchanger (NCX) is encoded by three genes; NCX1, 2 & 3. NCX1 has a variable region that is alternatively spliced in different tissues [15]. This variable region consists of 6 exons (termed A-F), the first two of which are mutually exclusive (figure 1C). NCX1.3, where the variable region consists of exons B and D only has previously been identified as the isoform present in HBSMC [16]. To date, NCX1.1 which is the isoform present in cardiac tissue has been the focus of most published research but there are clear differences between NCX1.1, expressed in excitable tissue and NCX1.3, expressed in non-excitable tissue (Hurtado *et al*., 2006).

The focus of this study was to define the functional role of NCX in cultured HBSMC. This included investigating the presence of transcript variants, exploring electrophysiological properties of reverse mode NCX currents, its contribution to agonist evoked Ca^2+ ^signals and airway contraction. In addition we show evidence of a link between SOC entry mediated by STIM1 and the activation of NCX1.

## Methods

### Ethical Approval

Human bronchial tissue was obtained from consenting patients with no history of asthma. Ethical approval was obtained from the Nottingham local ethical research committee.

### Human bronchial smooth muscle cells

Primary human bronchial smooth muscle cells (HBSMCs) were isolated from bronchial tissue by enzyme (collagenase) digestion and expanded through culture in Dulbecco's Modified Eagle Medium (DMEM) (containing 2 mM glutamine and 10% foetal calf serum (FCS)) using published methods [17]. Briefly, bronchial tissue was surgically removed from patients and placed in sterile, ice cold DMEM (without calcium or magnesium). The smooth muscle was removed from the surrounding epithelial and connective tissue and washed with DMEM. The smooth muscle was then finely chopped with a scalpel and incubated at 37°C with collagenase H (2 mg/ml) for 2-3 hours. Every hour, the cell mixture was filtered through a 100 μM cell strainer, pelleted by centrifugation and seeded into a 25 cm^3 ^culture flask. Cells were allowed to reach confluency before being passaged further. Cells between passage 3-6 were used in the experiments described in this study.

#### Transfection of siRNAs

NCX1 siRNA (AAGGAGAAGGAAATGAAACTG) and STIM1 siRNA (AAGGGAAGACCTCAATTACCA) were designed by and purchased from Ambion (Huntingdon, Cambridge, UK). A non-sense, scrambled siRNA was used as a negative control for transfection. Cells were transfected with 20 nM siRNA in FCS free medium over a period of 6 hours, the medium was then replaced with serum containing medium and cultured for a further 42 hours at 37°C, 5% CO_2_. The transfection reagent used was Lipofectamine 2000 (Invitrogen, Paisley, UK) at a final concentration of 1 μl/ml as per manufacturers' instructions.

#### Reverse transcriptase PCR

For reverse transcriptase (RT) PCR, primers against NCX2 and 3 were designed to span an intron-exon boundary (to eliminate genomic DNA contamination) using Primer 3 software [18]. Primer sequences are as follows; NCX2 forward:CATTCATGGAGGGAGCAGTT, NCX2 reverse: ATGACCAGGATGGAGACACC, NCX3 forward: AAGGTGCTGTTTGCCTGTGT, NCX3 reverse:TTTGCTGGCAAACGTATCTG. Primers for NCX1 (forward: TGTGAGTGAGAGCATTGGC, reverse: CTCTTTGCTGGTCAGTGGCT) were designed by Pitt *et al *[16] to span the alternatively spliced region and distinguish between transcript variants. Cycling was performed 35 times; 94°C, followed by 55°C (annealing temperature), then 72°C (all for 90 seconds) followed by 10 mins at 72°C. PCR products were visualized by ethidium bromide staining and confirmed by direct sequencing.

### Real-Time PCR (Taqman)

siRNA targeted mRNA knockdown was measured using real time, quantitative PCR (Taqman). Gene specific primers and probes against NCX1 were designed to span an intron-exon boundary using Beacon designer 7 (Stratagene): Forward primer; GCCTACTGACAGCTTTCATTGG, Reverse primer; TGTGTCTGGCACTGATGTTCC, probe; TGGCTTCCCACTTTGGCTGCACCA. 18 s RNA was used as a housekeeping gene to correct for equal cDNA input. The NCX1 probe was dual labelled with 5' FAM and 3'TAMRA dyes and was purchased from Applied Biosystems (Foster City, CA) along with the universal mastermix and the 18 s pre-designed assay. Each sample was run in duplicate and mRNA knockdown was measured from mRNA obtained from 3 separate experiments. The relative expression of the target gene was calculated using the comparative method (2^-ΔΔCt^) [19].

#### Immunostaining

HBSMC grown on cover slips were fixed with 4% formaldehyde and semi-permeabilized with 0.1% saponin as described [20]. Cells were incubated with mouse NCX1 antibody (1:200) (Abcam, Cambridge, UK) followed by labeling with anti-mouse Alexa fluor 488 (1:400) (Molecular probes). Cells were visualized on a spinning disk confocal microscope using a Zeiss Axio Observer D1, Hamamatsu electron multiplier CCD camera C0100-13 with a Yokogawa spinning disk system.

#### Measurement of Intracellular Calcium

Intracellular Ca^2+ ^levels ([Ca^2+^]_i_) in confluent, monolayers of HBSMC was estimated using Fluo-4AM. Cells were plated in 48 well plates and loaded with Fluo-4AM (Molecular probes) for 1 hour at room temperature in DMEM supplemented with 10% FCS, 2 mM glutamine and 2.5 mM probenecid (Sigma Chemical Co, Poole, UK). After loading, cells were washed with hanks balanced saline solution (HBSS) containing 10 mM HEPES, 2.5 mM probenecid, 2 mM CaCl_2 _and 1 mM MgCl_2_. After a 10 second baseline, cells were stimulated with either bradykinin (BK,1 μM) or histamine (HA,10 μM) for a total time period of 2 minutes. 10 μM KB-R7943 or equivalent DMSO vehicle control was added to the cells shortly before assay. Ca^2+ ^imaging was performed on the spinning disk confocal microscope system. Confocal images (1 image/second) were collected at 525 nm emission after excitation at 491 nm. Volocity analysis software (Improvision) was used to analyze the images. Each experiment incorporated at least 5 repeat wells per condition and each experiment was repeated at least 4 times. Data are presented as changes in mean fluorescence intensity (FI) compared with the baseline and peak fluorescence values was used as an estimation of changes in [Ca^2+^]i.

#### Patch-Clamp Electrophysiology

**Figure 2:**
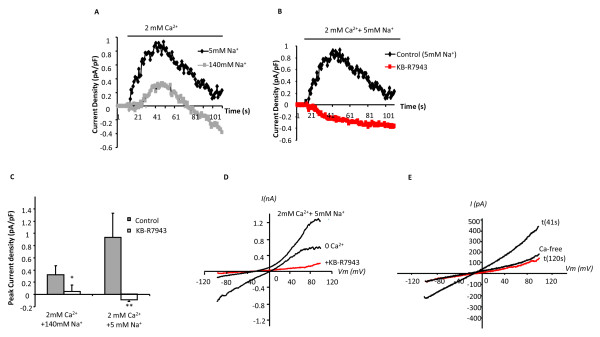
**Induction of KB-R7943 sensitive NCX1 outwardly rectifying currents under basal conditions**. (A)Time course of subtracted outwardly rectifying current density (+80 mV), each point represents mean data; 140 mM extracellular Na^+ ^(squares, n = 12) and 5 mM Na^+ ^(diamonds, n = 15). (B)Time course of subtracted current density showing control cells (diamonds, n = 15) compared with addition of KB-R7943 (squares, n = 6). (C) A bar chart illustrating peak current density of these outwardly rectifying currents (mean data ± SEM *p < 0.05, **p < 0.01) (D) Raw values (non-subtracted) for current-voltage (I-V) relationships at 0 extracellular Ca^2+^, following application of 2 mM extracellular Ca^2+ ^and reduced Na+ (5 mM) with or without KB-R7943. Note that in panel 2A and 2B baseline currents (zero Ca^2+^) have been subtracted from all traces. (E) Raw values (non-subtracted) for current-voltage (I-V) relationships at 0 extracellular Ca^2+^, following addition of 2 mM extracellular Ca^2+ ^and reduced Na+ (5 mM) at time points of 41 seconds (peak current) and 120 seconds (end point).

The conventional whole-cell patch-clamp technique [21] was employed to record NCX currents in single HBSMCs with an EPC-10 double amplifier and Patchmaster version 2.10 software (HEKA, Lambrecht, Germany). The outward exchange currents were activated by switching the external solution from the one containing no added Ca^2+ ^to the one containing 2 mM Ca^2+ ^with or without reduced 5 mM Na^+ ^as previously described [14]. SOCC was activated by the store-depletion agent-cyclopiazonic acid (CPA) dissolved in Ca^2+^-free solution as previously described [2,3]. The external solution contained: 140 mM NaCl, 1 mM MgCl_2_, 0 mM or 2 mM CaCl_2_, 10 μM nifedipine, 20 μM ouabain, 2 μM ryanodine, 100 μM niflumic acid and 5 mM HEPES (pH = 7.4). The electrode solution contained: 120 mM Cs-methanesulfonate, 20 or 40 mM NaCl, 5 mM MgCl_2_, 0.1 mM EGTA, 10 mM HEPES (pH = 7.2 adjusted with cesium hydroxide and methanesulfonic acid). The intracellular free Ca^2+ ^concentration (calculated by fura-2 ratiometric measurement) was 14 nM. Pipettes were drawn from borosilicate glass and had a resistance of 5-8MΩ when filled with internal solution. Average junction potentials of ~-10 mV between bath solution and electrode solution were generated and corrected before data acquisition by use of Patchmaster. Experimental drugs were delivered through a pressurised puffer pipette positioned 50 μm around the cells followed by a separate pressurised pipette used to wash out drugs (modified DAD-8 Macro Manifold perfusion system, reported time for switching between solutions ~12 ms). The bath solution was gravity perfused constantly from a 50 ml syringe at a flow rate of ~1 ml/minute. Cells were held at a membrane potential of -40 mV and current-voltage relationships were analyzed every 1 s from voltage ramps from -100 to +100 mV (1 Hz) at a rate of 0.5 V·s^-1^. Currents were filtered at 1 kHz and sampled at 4 KHz. Capacitance and series resistance readings were continuously monitored and the recording was discarded if a significant change occurred in either parameter. Current density at -80 mV and +80 mV were calculated and plotted against time following re-addition of Ca^2+^. By convention, positive (outward) currents taken at +80 mV reflect reverse-mode NCX-1 currents as the stoichiometry of this exchanger is 3Na^+^:1Ca^2+ ^[14,22,23]. Negative (inward) currents taken at -80 mV reflect SOCC. In most experiments outward currents taken at +80 mV in Ca^2+^-free solution were subtracted in all groups for the purpose of comparison between groups (as stated in figure legends). Measurements were taken at 41 seconds unless otherwise stated (see Figure 2 legend for further details).

#### Porcine lung slices

Whole porcine lungs were obtained from a local abattoir (Nottingham). An isolated, distal lobe was inflated to ~90% capacity with 2% agarose (Type VII, Sigma) solution (in HBSS) by inserting a tube via the trachea; the airways were cleared of agarose by injecting a pulse of air. Slices were cut ~130 μM thick using a vibrotome tissue slicer (EMS-4000, Electron Microscope Sciences). Lung slices were transferred to DMEM, supplemented with 2 mM L-glutamine and penicillin-streptomycin and incubated for 2~3 days at 37°C/5% CO_2_. Phase contrast images were recorded at a rate of 1 image per second. The difference in brightness between the airway lumen and surrounding alveolar tissue was used to detect the airway edge and calculate the area of the airway. Airway narrowing was expressed as the percentage decrease in airway area compared to the area of the fully relaxed airway (prior to agonist stimulation).

#### Statistical analysis

Unless otherwise stated, statistical significance between groups was assessed by unpaired T tests where *p < 0.05, **p < 0.01 were considered significant.

## Results

### NCX1 is expressed in HBSMCs

RT-PCR revealed that only NCX1 was present in HBSMCs (Figure 1A). Human brain cDNA was used as a positive control [13] to validate primers designed for NCX2 and NCX3. The NCX1 primers span the variable region of NCX1 and so distinguish between splice variants (designed by Pitt and colleagues [16]) (Figure 1C). RT-PCR gave a single PCR product (Figure 1A) and sequence analysis revealed that within the variable region, only exons B and D were present. This was repeated for three different donors. Therefore the isoform that we found in HBSMCs was NCX1.3 and is the same variant that has previously been found in this tissue type [16]. In addition, positive staining of NCX1 protein was found in permeabilised HBSMCs (Figure 1B). A Negative control sample where only secondary antibody was applied revealed no positive staining (data not shown).

### Reverse mode NCX currents are induced in HBSMCs using ion replacement experimental conditions and are augmented by stimulation with histamine

Outwardly rectifying or reverse mode NCX1 currents in HBSMCs were recorded using the modified whole cell voltage clamp technique as previously described [14,22,23].

The cells were initially perfused with a Ca^2+^-free extracellular solution containing 140 mM Na^+^. Extracellular Ca^2+ ^was applied to the Na^+ ^loaded cell along with physiological (140 mM) or reduced (5 mM) Na^+ ^to induce an outwardly rectifying current mediated by reverse mode NCX1 i.e. 3Na^+ ^efflux and 1Ca^2+ ^influx according to the stoichiometry of this exchanger (3Na^+^:1Ca^2+^). The intracellular solution was buffered to low Ca^2+ ^(~14 nM), and high Na^+ ^(20 mM) which favors reverse mode NCX1. Under these conditions, perfusion with Ca^2+ ^free extracellular solution with 140 mM Na^+ ^induced a current, with a linear current-voltage relationship and a reversing potential of ~ 0 mV (Figure 2D). The characteristics of this current resemble that of SOC channels. Re-addition of 2 mM extracellular Ca^2+ ^reduced these currents and subsequently activated a small transient outwardly rectifying current with a reversal potential of -22.56 ± 4.67 mV (n = 14). A typical example of the current activated by extracellular application of 2 mM Ca^2+ ^is illustrated in Figure 2A. This outwardly rectifying current was abolished by 10 μM KB-R7943 (Figure 2B-D). Furthermore, when extracellular Na^+ ^was reduced from 140 mM to 5 mM (along with re-addition of 2 mM extracellular Ca^2+^), the outwardly rectifying current was significantly augmented. The peak current density following extracellular 2 mM Ca^2+ ^re-addition with 5 mM Na^+ ^was increased by 243% compared to that with 140 mM Na^+^. This increase in outwardly rectifying current was also abolished by 10 μM KB-R7943 (Figure 2A). These results suggest that NCX1 is present in cultured HBSMCs and is activated upon these specific changes in experimental conditions.

**Figure 3:**
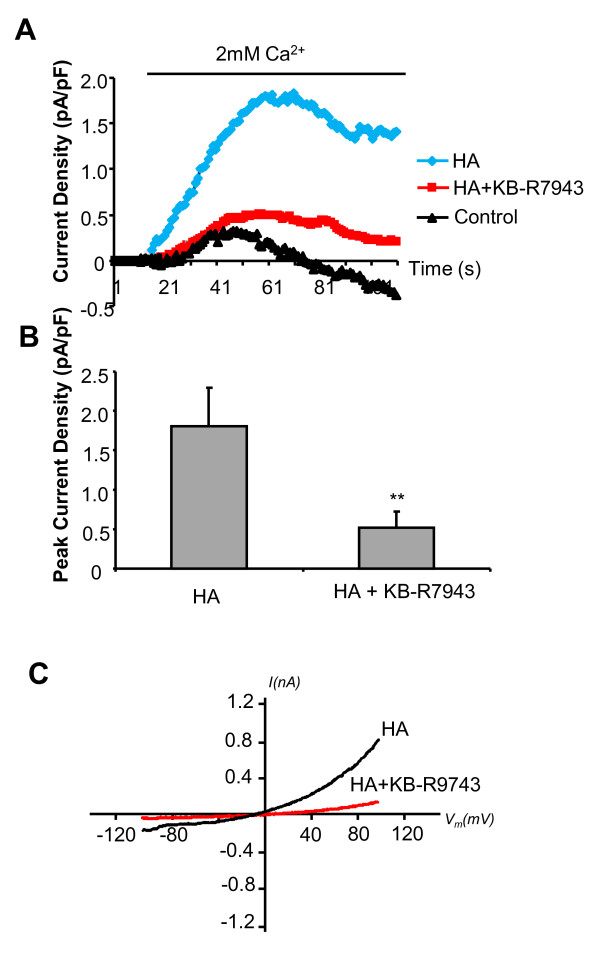
**10 μM Histamine (HA) increased NCX1 mediated outwardly rectifying currents and are inhibited by 10 μM KB-R7943**. (A) Time course of current density (at +80 mV). Control (triangles, n = 12), HA (diamonds, n = 10) and HA+KB-R7943 (squares, n = 13). (B)Bar chart illustrating peak current density of these HA induced outwardly rectifying currents with (n = 13) or without KB-R7943 (n = 10) mean data ± SEM, **p < 0.01. C) Representative traces of I-V relationships of currents induced by re-addition of extracellular Ca^2+ ^and HA ± KB-R7943. Note that in panel 3A baseline currents (0 Ca^2+^) have been subtracted from all traces.

To assess the effect of HA on basal NCX1 currents, 10 μM HA was added along with the re-addition of 2 mM extracellular Ca^2+ ^(with 140 mM Na^+^). Outwardly rectifying currents following re-addition of extracellular Ca^2+ ^were markedly enhanced in the presence of HA (Figure 3A). The augmented outwardly rectifying current induced by HA displayed an I-V relationship that is characteristic of reverse mode NCX1 (Figure 3C). In addition this current was significantly inhibited by 10 μM KB-R7943 (Figure 3B). These results suggest that cellular stimulation with HA results in the increased activation of an outwardly rectifying current that is characteristic of reverse mode NCX1.

### Knockdown of NCX1 and STIM1 by siRNA results in reduction of histamine induced outwardly rectifying currents

**Figure 4:**
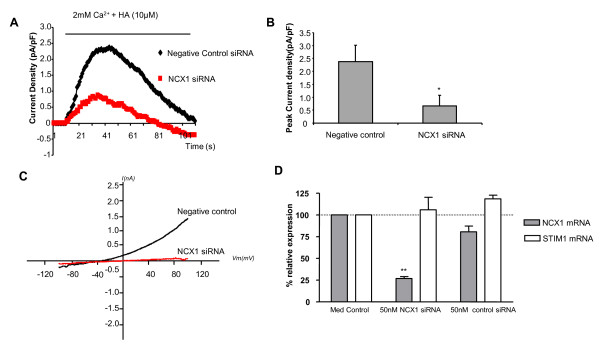
**HBSMCs transfected with NCX1 siRNA have reduced NCX1 expression and display reduced HA mediated NCX1 outwardly rectifying currents**. Reverse mode NCX1 was activated by re-addition of 2 mM extracellular Ca^2+ ^and 10 μM HA. (A) Time course of NCX1 outward current density (at +80 mV) obtained by subtracting the current recorded in Ca^2+^-free solution; Control (diamonds, n = 13) and NCX1 siRNA (squares, n = 17). (B) Bar chart illustrating peak current density of NCX1 outwardly rectifying currents in cells transfected with either negative control siRNA (n = 13) or NCX1 siRNA (n = 17) (mean data ± SEM, *p < 0.05). C) Representative traces of current-voltage (I-V) relationships of NCX1 currents in cells transfected with control or NCX1 siRNA. D) siRNA targeted knockdown of NCX1 mRNA assessed by quantitative PCR. Cells transfected with 50 nM NCX1 siRNA reduced NCX1 mRNA levels (73.2 ± 2.2%, n = 4) with no effect on STIM1 expression. Note that in panel 4A baseline currents (0 Ca^2+^) have been subtracted from all traces.

KB-R7943 has proven to be a valuable tool in the study of NCX1. However, selectivity issues of KB-R7943 for NCX1 over other ion channels have been previously reported [24,25]. Therefore, to confirm that the outwardly rectifying currents induced by HA were mediated by NCX1, we selectively knocked down NCX1 using target specific siRNA. HBSMCs were transfected with 50 nM NCX1 siRNA or 50 nM negative control (a non-sense siRNA sequence). Experiments were performed 48 hours following transfection. The effectiveness of NCX1 siRNA was assessed by real-time PCR (taqman). Transfection of 50 nM NCX1 siRNA resulted in a 73.2 ± 2.2% (n = 4) reduction of NCX1 mRNA (see Figure 4D). No reduction of STIM1 mRNA was observed following transfection of 50 nM NCX1 siRNA. Validation of STIM1 siRNA transfection has been previously described [2]. Transfection of 20 nM STIM1 siRNA resulted in a 76.9 ± 7.5% reduction in STIM1 mRNA levels (data not shown). Further details can be found in a previous manuscript [2]. Reverse mode NCX1 currents induced by 10 μM HA were compared between NCX1 siRNA transfected cells and equivalent negative control transfected cells. The results are shown in Figure 4. 17 cells were studied in the NCX1 siRNA group compared with 13 cells analyzed in the negative control group. Of the 17 cells in NCX1 siRNA group, the NCX1 outwardly rectifying currents in 9 cells were completely abolished. In the remaining 8 cells, 6 cells showed reduced outwardly rectifying currents compared with negative control. Of the 13 cells transfected with negative control, 12 cells exhibited a robust NCX1 outwardly rectifying current, 1 cell displayed no detectable outwardly rectifying current. Overall peak current density in the entire NCX1 siRNA group was decreased by 63.4% (0.87 ± 0.46 compared with 2.37 ± 0.65 pA/pF in the negative control group, p < 0.05) (Figure 4A-C). Thus we concluded that NCX1 siRNA transfection effectively blocked reverse mode NCX1 currents. NCX1 siRNA had no significant direct effect on SOC (data not shown).

**Figure 5:**
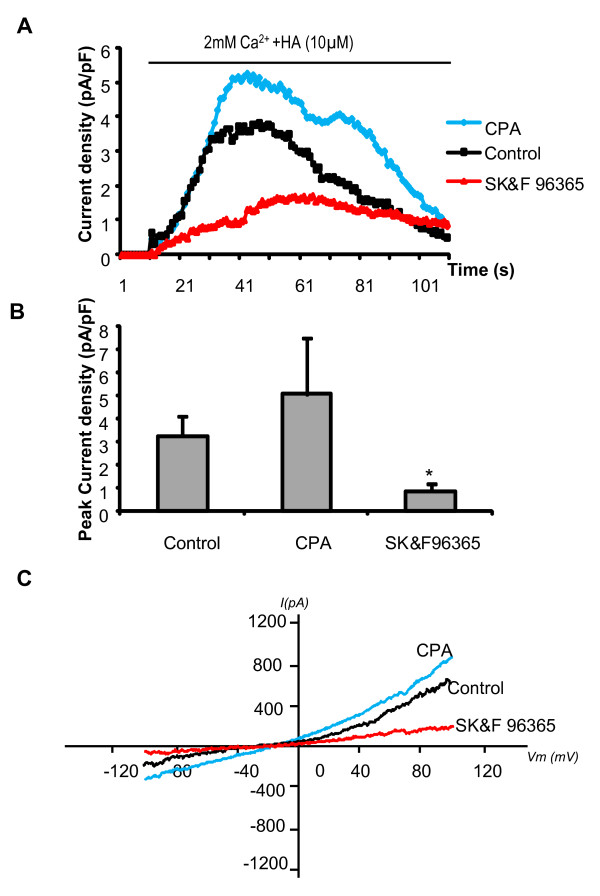
**Effect of 10 μM CPA and 20 μM SK&F 96365 on HA induced NCX1 currents in HBSMCs**. (A) Time course of NCX1 subtracted outwardly rectifying current density (at +80 mV) ± CPA or SK&F 96365. Control (squares, n = 11), CPA (diamonds, n = 12) and SK&F 96365 (triangles, n = 11). B) Bar chart illustrating peak current density of NCX1 outwardly rectifying current in control (n = 11) compared with cells treated with CPA (n = 12) or SK&F 96365 (n = 11) (mean data ± SEM, *p < 0.05). C) Representative traces of I-V relationships of NCX1 currents ± CPA or SK&F 96365.

These results demonstrate that an outwardly rectifying current mediated by NCX1 is activated by HA in cultured HBSMCs. We next tested the hypothesis that Na^+ ^influx via SOC channels is involved in the mechanism of induction of reverse mode NCX1 following HA simulation. We have previously shown that store depletion by cyclopiazonic acid (CPA) activates a non-selective current in HBSMCs which is mediated by STIM1 [2]. Indeed, the NCX1 mediated outwardly rectifying current that we describe in this current study is preceded by a SOC like current and is inhibited by SK&F 96365, a known inhibitor of SOC channels (Figure 5A-C). Stimulation with 10 μM CPA only marginally enhanced the NCX1 current. CPA is an inhibitor of the SR Ca^2+^/ATPase and mediates passive depletion of intracellular store Ca^2+^. However, the protocol we describe in this present study includes a prolonged perfusion with Ca^2+ ^free extracellular buffer in the presence of ryanodine and so the intracellular stores are likely to be already depleted. This may go some way to explain why the inclusion of CPA did not significantly enhance the NCX1 outwardly rectifying current proposed to be mediated by Na^+ ^influx through SOC channels.

**Figure 6:**
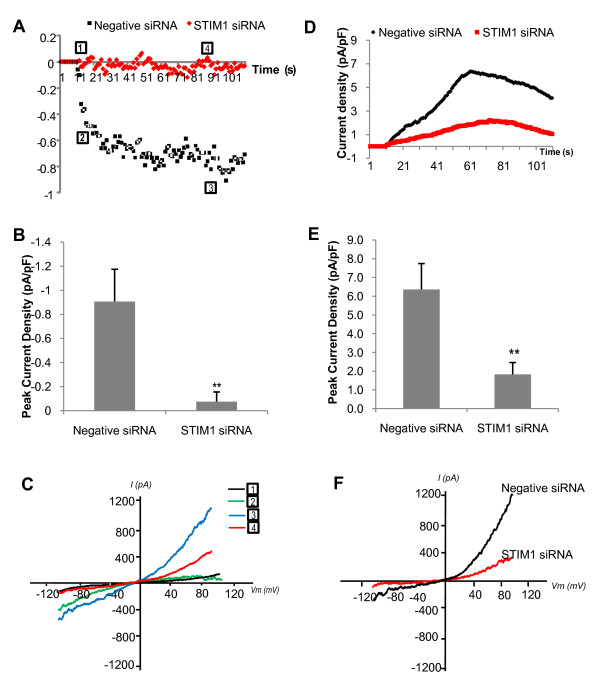
**STIM1 knockdown abolished SOC currents**. Na^+ ^concentration in the electrode solution has been increased to 40 mM while Na^+ ^concentration in the extracellular solution remains the same (140 mM) A)Time course of SOC current density (at -80 mV) in cells transfected with negative control siRNA (squares, n = 17) or STIM1 siRNA (diamonds, n = 17). B) Bar chart illustrating peak current density of SOC current in control siRNA transfected cells compared with cells transfected with STIM1 siRNA (mean data ± SEM, **p < 0.01). C) Representative traces of I-V relationships of SOC currents. Numbers 1-4 correspond to time points on time course chart A. D) Time course of NCX1 outwardly rectifying current density (at +80 mV) in cells transfected with negative control siRNA (diamonds, n = 17) or STIM1 siRNA (squares, n = 17). E) Bar chart illustrating peak current density of NCX1 outwardly rectifying current in control siRNA transfected cells compared with cells transfected with STIM1 siRNA; mean data ± SEM, **p < 0.01. F) Representative traces of I-V relationships of NCX1 outwardly rectifying currents.

However, combined perfusion with a Ca^2+ ^free extracellular buffer and the addition of HA induced a Na^+ ^mediated current which was completely abolished following siRNA mediated knockdown of STIM1 (Figure 6A-C). Interestingly, the NCX1 mediated current which is induced shortly after this SOC current was also greatly inhibited following STIM1 knockdown (Figure 6D-F). These results provide the first evidence for a link between SOC associated currents mediated by STIM1 and the activation of NCX1.

### KB-R7943 partially inhibits agonist evoked Ca^2+^signals in HBSMCs

**Figure 7:**
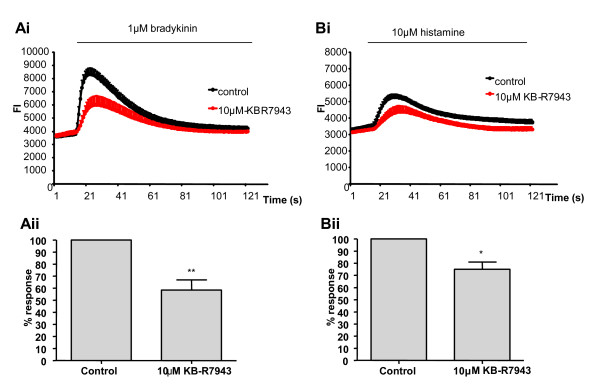
**Inhibition of reverse-mode NCX1 by KB-R7943 results in decreased agonist induced Ca^2+ ^signals**. (Ai) Representative experimental trace from one experiment (average of 5 wells) illustrating BK (1 μM) induced changes in [Ca^2+^]_i _(presented as fluorescence intensity (FI)). (Aii) Summary of the data illustrated in (Ai) showing averaged peak fluorescence data, following addition of BK (n = 6). (Bi) Representative experimental trace from one experiment (average of 5 wells) illustrating HA (10 μM) induced changes in [Ca^2+^]_i _(ii). Summary of the data illustrated in (Ai) showing averaged peak fluorescence data, following addition of HA (n = 4). Summary graphs (Ai &Bi) are expressed as % changes ± sem compared to control and represent averaged data from at least 4 different experiments. Statistical analysis (paired Student T Test) was performed on the raw data and are indicated as significant with *P < 0.05 and **P < 0.01.

We next investigated the effects of 10 μM KB-R7943 on Ca^2+ ^signals in cultured HBSMCs induced by both 1 μM BK and 10 μM HA. The results are shown in Figure 7. When bathed in HBSS containing 2 mM Ca^2+^, both BK and HA induced an elevation of intracellular Ca^2+ ^in cultured HBSMCs. The response is biphasic and consists of a large rapid increase in Ca^2+ ^which peaks within seconds of agonist stimulation and is followed by a lower but more sustained elevation of Ca^2+^. When pre-incubated with 10 μM KB-R7943 (for 1 minute prior to agonist stimulation), HBSMCs respond to both BK and HA with a reduced intracellular Ca^2+ ^signal. Peak BK induced fluorescence signals were reduced by 41% (n = 6) and peak HA signals were reduced by 25% (n = 4).

### KB-R7943 relaxes pre-contracted porcine airways

**Figure 8:**
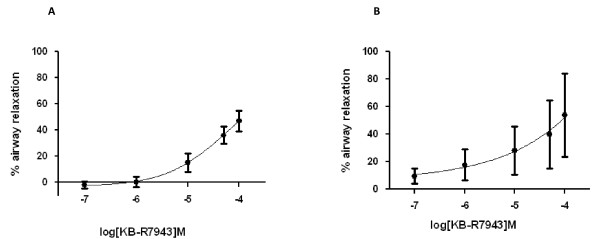
**KB-R7943 dose dependently relaxes pre-contracted porcine small airways**. Averaged responses (n = 4 airways, 1 pig) of % relaxation induced by increasing doses of KB-R7943 (from airways pre-contracted with 1 μM ACh (panel A) or 10 μM HA (panel B). % relaxation was assessed by measuring the luminal area of the airway, normalised against the area of the fully relaxed airway (pre-stimulation) and contraction induced to the agonist. HA induced a contractile response which was 62.73 + 13.38% of that observed with ACh.

The effects of KB-R7943 on porcine airway contraction were next investigated. Lung slices were prepared as described in the methods section. Small peripheral airways (approximately 150 μm in diameter) with visibly beating cilia were selected for experimentation. After establishing a baseline where the airway was fully relaxed, 1 μM ACh or 10 μM HA was perfused over the lung slice to induce a sustained contraction. Following this, increasing additive concentrations of KB-R7943 (0.1 μM -100 μM), in the continual presence of agonist was perfused over the slice. KB-R7943 dose dependently relaxed the pre-contracted airways, with a noticeable relaxation occurring from 10 μM KB-R7943. Figure 8 shows averaged data (n = 4) for % relaxation with both agonists. Values from the last 10 seconds of each concentration were averaged. Time matched DMSO controls were performed to ensure the airway could remain fully contracted for the duration of the experiment (data not shown).

## Discussion

This present study provides, for the first time, molecular and electrophysiological data as well as cellular and tissue pharmacological evidence supporting the presence of a functional NCX1 protein that can operate in reverse mode and thus presents an additional mechanism of Ca^2+ ^influx in HBSMCs. We also provide additional evidence in support of an existing hypothesis whereby Na^+ ^influx through SOC channels activates reverse mode NCX; to our knowledge we are the first to show a link between STIM1 mediated Na^+ ^influx and the activation of NCX1.

We initially confirmed expression of NCX1 mRNA and protein in HBSMCs. The splice variant we identified was NCX1.3 and is consistent with previous findings [16]. We could not detect the presence of other NCX1 splice variants or the expression of NCX2 or NCX3 in this cell type. NCX1 (weak band) NCX2 and NCX3 expression was detected in human brain and served as a positive control for these primer pairs. Expression of NCX1 protein was also confirmed by immunohistochemistry using specific NCX1 antibodies. We found that NCX1 protein was expressed both on the membrane and in the cytoplasm of the cells. There is some evidence of membrane bound NCX1 protein being trafficked into the cytoplasm which could explain this result [26].

Following confirmation of the expression of NCX1.3 in cultured HBSMCs, we then studied the functional role of this protein and its contribution to agonist evoked Ca^2+ ^signals in these cells. NCX1.1 is the splice variant expressed in cardiomyocytes and has been extensively studied and its electrophysiological properties are well characterized [27]. Few studies have been performed in cells expressing NCX1.3; therefore we firstly aimed to characterize the currents carried by NCX1.3 in HBSMCs using whole cell patch clamp.

Experimental conditions were adjusted to favour the activation of reverse mode NCX1 currents. Cells were first loaded with Na^+ ^through perfusion with a Ca^2+ ^free extracellular buffer and the addition of ouabain. Reverse mode NCX1 was subsequently activated via re-addition of 2 mM extracellular Ca^2+^. The outwardly rectifying current displayed typical characteristics to that of Na^+ ^efflux through NCX1 and were inhibited by KB-R7943 (a relatively selective inhibitor of reverse mode NCX1). These currents were greatly enhanced by stimulation with the contractile agonist HA and these HA enhanced currents were inhibited by KB-R7943. However, because at high concentrations KB-R7943 can have effects on other channels, we also used knockdown of NCX1 by siRNA, with similar results. Perfusion with Ca^2+ ^free extracellular buffer activates a SOC like current prior to the activation of the NCX1 mediated outwardly rectifying current. Interestingly, inhibition of this SOC current by prior knockdown of STIM1 also inhibited HA induced NCX1 currents. As far as we are aware, these are the first data that provide evidence of a functional link between STIM1 mediated SOC activation and reverse mode NCX1 activation.

The electrophysiology data provided evidence of a functionally active NCX1 that is activated following HA induced SOC activation. We then further tested its physiological relevance through measurement of agonist induced Ca^2+ ^signals and contraction of ASM. The addition of 10 μM KB-R7943 almost completely abolished NCX1 sensitive currents. However, in comparison the equivalent concentration of inhibitor only marginally inhibited HA induced Ca^2+ ^signals. The experimental conditions used to yield the electrophysiology results were designed to optimise and isolate NCX1 currents. Experimental conditions implemented in Figure 7 were under more physiological conditions and therefore a range of other Ca^2+ ^influx pathways (insensitive to NCX1 inhibition) would have been activated. Inhibition of reverse mode NCX1 by KB-R7943 resulted in dose dependent relaxation of pre-contracted small airways, illustrating the functional importance of NCX1 in maintaining contraction. Addition of KB-R7943 also inhibited both BK and HA induced intracellular Ca^2+ ^signals in HBSMCs.

Abnormal Ca^2+ ^handling within ASM is likely to contribute to inappropriate contraction, a major symptom of asthma. The understanding of how intracellular Ca^2+ ^is regulated under physiological and pathophysiological situations forms an important aspect in the search for new therapeutic targets for the treatment of asthma. In recent years, there have been major advances in the understanding of Ca^2+ ^homeostasis driven in part by the identification of STIM1 and ORAI1 as critical regulators of Ca^2+ ^influx in many tissue types. In HBSMCs, we have shown that the interaction between STIM1 and ORAI1 plays an important role in the activation of SOC channels [2,3]. The precise molecular identity of these SOC channels is still under debate with strong evidence for both ORAI and TRPC homologues. Our data supports the hypothesis whereby histamine (HA) stimulation results in IP_3 _mediated Ca^2+ ^store release, resulting in store depletion of Ca^2+^. This in turn activates SOC channels via a STIM1 dependent mechanism. Activation of SOC channels permits influx of Na^+ ^(plus a small amount of Ca^2+^). This localized increase of intracellular Na^+ ^results in membrane depolarization that activates reverse mode NCX1 to further promote Ca^2+ ^influx.

In summary, we provide evidence demonstrating that reverse mode NCX1 plays a role in the control of Ca^2+ ^homeostasis in ASM. This role is dependent on STIM1 activation of SOC following intracellular SOC depletion. Whilst other Ca^2+ ^entry mechanisms may also play a part in Ca^2+ ^influx, these data suggest targeting NCX may provide a novel therapeutic strategy in diseases such as asthma.

## Abbreviations

ASM: airway smooth muscle; BK: bradykinin; FCS: foetal calf serum; HA: histamine; HBSMC: human bronchial smooth muscle cells; ROC: receptor operated cation; SOC: store operated cation; SR: sarcoplasmic reticulum.

## Competing interests

The authors declare that they have no competing interests.

## Authors' contributions

BL and SP designed research, performed experiments and wrote the paper, JF performed lung slice experiments and IH supervised, contributed to experimental design and data interpretation. All authors read and approved of the final manuscript.
